# Missing body language

**DOI:** 10.1093/jhps/hnaa066

**Published:** 2020-12-27

**Authors:** Richard (Ricky) Villar

**Affiliations:** Editor-in-Chief Journal of Hip Preservation Surgery

The other day I heard a colleague say how strange it was that the once unusual event had now become a daily occurrence. He was talking of the seemingly endless webinars that the pandemic forces us to attend online. I now go to meetings, when I mean I sit in front of a screen. I make sure my top half is spick and span, dressed for the occasion, while my bottom half, out of sight, is something completely different. The new normal is the webinar, where we try to feel part of a team, yet sit alone in an office, study, bedroom, wherever we have decided to make our workspace. When I join my digital colleagues, I may be talking to them, but I am also looking at where they are seated. What’s that on the wall behind? A family photo, a rosette from a dressage competition, or perhaps a fine work of art. Or is my colleague forced to make a brave face as he squats on an upstairs landing, while in the distance I hear his children creating mayhem? In the early stages of the pandemic, I felt that webinars were temporary, that soon we would be back to how it was. Sadly, that is not the case, and for the present, there seems little sign of things improving.

There are many who say that online meetings are here to stay, and I agree they have their uses. But there is something missing. Body language for sure, that look in the eye, the reaction to an unexpected statement, little features that may seem unimportant but mean so much. Virtual meetings may have lower cost, less environmental impact, greater and more international attendance, be speedier and be simple to record and distribute. They are also simpler to measure—attendance numbers, time spent online, polling counts and more. Meanwhile face-to-face (F2F) events, which was the way of things 12 months ago, take people to a more focused environment with fewer distractions. There is opportunity for brainstorming, networking and relationship building that the digital world simply cannot offer. For the trade, what better way is there to target your product than to attend a F2F meeting with a large cohort of folk who are genuinely interested in what you can offer? There are obvious costs, as F2F can be expensive—travel, room hire, catering, audiovisual support and so much more [1].

But I do miss the body language, which has helped me reach decisions for many years. These days all I see is a face, and on a computer screen that is hard to interpret. A smile suggests either approval or happiness. A frown can be the opposite [2]. The most trustworthy facial expression is a slight raise of the eyebrows with an added smile, something that conveys friendliness and confidence [3]. Narrower faces and prominent noses are said to reflect high intelligence and people who are smiling are reported to be cleverer [4]. Rapid blinking shows distress or discomfort while infrequent blinking suggests that a person is trying to control their eye movements [5].

In pre-pandemic days, if someone looked at me while either of us was talking, I felt I had their attention. Breaking eye contact suggested that they were distracted, uncomfortable or trying to conceal their feelings [6]. Yet I am now frequently addressed by a colleague who is looking anywhere but at me. My image may be somewhere on their screen, and they may be looking at my image as they talk, but they are not looking at me. 

Body movements and posture have now gone, as have touch, personal space and tiny gestures. Each is a different type of non-verbal communication [7]. I am left largely with the tone of voice, which is often fuzzy thanks to poor reception. How I crave the day when this so-called new normal of digital communication settles down and F2F is re-established.

Turning to our journal, this journal, JHPS, I was more than excited by the last issue, number 7.2. It took ages to be published, I was told thanks to Covid-19, but once it had appeared, its content was tremendous. As you might expect, I have read it from cover to cover, so to select any one paper is impossible. I was, however, especially fascinated by Lindman *et al.*’s paper on loss to follow-up [8]. I had always thought that non-responders on a registry were likely to have done worse. Apparently not. The outcome at 2 years after hip arthroscopic surgery was the same, for responders and non-responders, although responders ended up more satisfied. I was also spellbound by Chen *et al.* [9], as they discussed the evolution of arthroscopic surgery of iliopsoas. I remember the very early days of hip arthroscopy when we thought that even to see iliopsoas was a success. Then came the era of tenotomies, which in due course became less popular. In Chen *et al.*’s paper, it appears that although 75% of surgeons were less enthusiastic about iliopsoas tenotomy because of the weakness of hip flexion, they could not cite any published evidence to support their decision. It was the perception of poor outcome that changed their views. Sadly, so much of what we do is based on perception, even if there are journals such as *JHPS* trying to reverse that.

As for this issue, number 7.3, once again our journal is filled with excellence. It is still impossible for me to choose a favourite paper. Nor should I do so. I did find the paper by Sobti *et al.* [10] particularly fascinating, as they established that biological chondral reconstruction had a better outcome than microfracture. Their microfracture group had a much higher failure rate than patients who underwent biological reconstruction. In this latter group, 100% were going strong at 18 months after surgery. Impressive.

Another paper that caught my eye was that by Smith *et al.* [11], who suggested that fellowship training flattened the learning curve for periacetabular osteotomy. Obvious? Perhaps so, but to have fellowships supported academically is valuable, especially in parts of the world where healthcare systems may question a surgeon’s desire to spend 6 to 12 months overseas, learning something different. I am sure this article’s findings can be applied to many techniques, not just periacetabular osteotomy (PAO).

So, as ever, please enjoy this issue of *JHPS*. It is published for you, the hip preservation practitioner, and is filled from cover to cover with brilliance. I commend this issue to you in its entirety.

Oh yes, and please read, use and cite this journal at every opportunity. Ask everyone you know to do the same.

My very best wishes to you all.
